# Occupational Therapy Innovations in Home and Community Practice for People Living With Dementia

**DOI:** 10.1093/geroni/igaa057.2766

**Published:** 2020-12-16

**Authors:** Catherine Piersol, Scott Trudeau

**Affiliations:** 1 Thomas Jefferson University, Ardmore, Pennsylvania, United States; 2 American Occupational Therapy Association, Bethesda, Maryland, United States

## Abstract

Most Americans with dementia live at home and families are commonly responsible for overseeing the daily activities of the person with dementia. Families require support, education, and skill-building to manage caregiving responsibilities. Occupational therapist are ideally suited to teach care partners tailored strategies for obtaining the “just right fit” between the capacities of the person and the demands of the environment, thus reducing behavioral symptoms, optimizing function and safety and enhancing well-being. Care of Persons with Dementia in their Environments (COPE) is an evidence-based intervention designed to address these outcomes. Over three phases, the occupational therapist employs a problem-solving method to identify strategies that address caregiver-reported difficulties related to managing daily activities, behavioral challenges and other caregiver concerns. This presentation describes the development and implementation of COPE highlighting the distinct approaches of occupational therapy in delivering home- and community-based services to persons living with dementia and their care partners.

